# Combining therapy with recombinant human endostatin and cytotoxic agents for recurrent disseminated glioblastoma: a retrospective study

**DOI:** 10.1186/s12885-019-6467-6

**Published:** 2020-01-08

**Authors:** Jing-jing Ge, Cheng Li, Shao-pei Qi, Feng-jun Xue, Zhi-meng Gao, Chun-jiang Yu, Jun-ping Zhang

**Affiliations:** 1 Department of Neuro-Oncology, Sanbo Brain Hospital, Capital Medical University, No. 50, Yi-Ke-Song Road, Haidian District, Beijing, 100093 People’s Republic of China; 2 Department of Neurosurgery, Sanbo Brain Hospital, Capital Medical University, Beijing, 100093 China

**Keywords:** Recurrent disseminated glioblastoma, Recombinant human endostatin, Temozolomide, Irinotecan, Chemotherapy

## Abstract

**Background:**

The optimal chemotherapeutics of recurrent disseminated glioblastoma has yet to be determined. We analyzed the efficacy and safety of recombinant human endostatin (rh-ES) combined with temozolomide and irinotecan in patients with recurrent disseminated glioblastoma.

**Methods:**

We retrospectively reviewed 30 adult patients with recurrent disseminated glioblastoma treated with this combination chemotherapy at Department of Neuro-Oncology, Sanbo Brain Hospital, Capital Medical University of China from November 2009 to August 2018. Temozolomide was given orally at 200 mg/m^2^ daily for 5 days and rh-ES was administrated 15 mg/d daily for 14 days of each 28-day treatment cycle. Irinotecan was given intravenously every 2 weeks on a 28-day cycle at 340 mg/m^2^ or 125 mg/m^2^ depending on antiepileptic drugs. Primary endpoint was progression-free survival (PFS) at 6 months (6 m-PFS).

**Results:**

The 6 m-PFS was 23.3%. The median PFS was 3.2 months. The overall survival rate (OS) at 12 months was 28.6%. The median OS was 6.9 months. Six out of 30 (20%) patients demonstrated partial radiographic response and 11 (36.7%) remained stable. The PFS of the 6 patients who got partial response was 5.8, 6.3, 6.9, 13.6, 15.8 and 16.6 months, respectively, and the median time interval of first response was 4 (range, 2.0–6.6) months. The most common adverse events were hematologic toxicities and gastrointestinal effects. The Grade ≥ 3 adverse event was hematologic toxicities. The adverse events were manageable.

**Conclusions:**

Rh-ES, in combination with cytotoxic drugs, was an alternative effective regimen with manageable toxicities in treatment of recurrent disseminated glioblastoma.

## Background

Recurrent glioblastoma with extensive intracranial or spinal dissemination is refractory and have a poor prognosis. Re-irradiation is not always recommended due to the dose restriction, large tumor volume, tumor extensive locations, and late adverse events which included irreversible white matter changes and radionecrosis [1].

Chemotherapy is the primary treatment for those recurrent disseminated glioblastoma. However, there is no standard chemotherapy regimen currently. Anti-angiogenesis is a promising therapeutic strategy because that pathological angiogenesis is necessary for glioblastoma occurrence and metastasis. Bevacizumab, a humanized monoclonal antibody that inhibits vascular endothelial growth factor (VEGF), improved the progression-free survival (PFS), but had no effect on the overall survival (OS) [2–4]. Moreover, the effects of bevacizumab are transient and most patients’ tumors progress just after a median time of 3–5 months [5–7]. Recombinant human endostatin (rh-ES) is an endogenous broad-spectrum angiogenesis inhibitor that has been shown to significantly improve therapeutic efficacy when combining with conventional chemotherapy agents in non-small-cell lung cancer, breast cancer and melanoma [8–10]. A previous study reported a case with recurrent brainstem pilocytic astrocytoma with neuraxis dissemination achieved a long-term tumor remission (more than 29 months) after receiving rh-ES in combination with conventional chemotherapy regimen of vincristine and carboplatin [11]. However, there has been no clinical experience reported on the use of rh-ES in glioblastoma treatment to date.

In this study, we retrospectively analyzed the effect and toxicity of rh-ES when combined with traditional cytotoxic drugs on adult recurrent disseminated glioblastoma.

## Methods

### Patients selection

We performed a retrospective observational study for all adult patients with recurrent disseminated glioblastoma treated with the combined regimens of temozolomide (TMZ), irinotecan (CPT-11) and rh-ES at Department of Neuro-Oncology, Sanbo Brain Hospital, Capital Medical University from November 2009 to August 2018.

Inclusion criteria were as follows: Age at 18–70 years old; Histological diagnosis of glioblastoma, the glioblastoma secondary to low-grade gliomas were also included; Recurrence was confirmed histologically or by radiographic evidence after surgery and adjuvant radiotherapy; Intracranial and/or spinal cord disseminated lesions at recurrence, whether lesions were disseminated at diagnosis was not limited; Treated with at least one cycle of the combined chemotherapy (TMZ, CPT-11 and rh-ES); Had at least one post-treatment radiographic follow-up. Patients who had previously received anti-angiogenic drug were also considered eligible. Patients with incomplete medical records were excluded.

### Treatment

TMZ was given orally 200 mg/m^2^ for 5 days in each cycle. CPT-11 was administrated 125 mg/m^2^ for patients not receiving enzyme-inducing antiepileptic drugs (EIAEDs) and 340 mg/m^2^ for patients receiving EIAEDs on day 1 and day 15. Rh-ES was administrated 15 mg/d, daily for 14 days. One cycle of therapy was defined as 28 days. The clinical data collected included the following: Age, sex, previous therapies, number of relapse, treatment cycles, response, adverse events, subsequent treatments, progression date and dead date. The primary endpoint was PFS at 6 months (6 m-PFS). Secondary endpoints were median PFS, OS, OS at 12 months (12 m-OS), objective response rate (ORR), disease control rate (DCR) and adverse events.

### Method of evaluation

Contrast-enhanced MRI was performed at baseline and every 2 cycles thereafter until disease progression. Radiographic responses of the tumor were classified according to the RANO criteria [12]. A minimum of two largest lesions should be measured. For patients who have multiple lesions of which only one or two are increasing in size, the target enlarging lesion should be measured. Toxicities were classified according to Common Terminology Criteria for Adverse Events (CTCAE) 4.0.

### Statistical analysis

Continuous variables were described by median and range, whereas categorical variables were described by numbers and percentages. The PFS and OS were analyzed using non-parametric Kaplan-Meier method. All statistical analyses were performed using SPSS 17.0. *p* < 0.05 was considered to be statistically different.

## Results

### Patient characteristics

Between November 2009 and August 2018, 39 patients with recurrent glioblastoma were treated with the combined regimen. Excluding 9 patients whose tumors were not disseminated, 30 patients met the inclusion criteria with 21 men (70%) and 9 women (30%). The median age was 43 (range: 21–59). Patient characteristics are presented in Table 1. Five (16.7%) patients’ tumors were both intracranial and spinal disseminated and 14 (46.7%) recurred more than once. Fourteen (46.7%) patients received > 2 previous regimens and 5 (16.7%) received previous bevacizumab treatments. MGMT promoter was unmethylated in 9 patients and methylated in 4 patients. One patient harbored mutated IDH and 10 harbored wild-type IDH.

**Table 1:** Patient characteristics

Patient characteristics	r GBM, *n* = 30
Age, years	
Mean	41.8
Median	43
Range	21–59
Sex, n (%)	
Male	21 (70.0)
Female	9 (30.0)
Initial KPS, n (%)	
50–60	8 (26.7)
70–80	16 (53.3)
90–100	6 (20.0)
Relapse, n (%)	
First	16 (53.3)
Second	7 (23.3)
Third	7 (23.3)
Resection at last relapse before enrollment, n (%)	
Yes	3 (10.0)
No	27 (90.0)
Previous radiotherapy, n (%)	
Yes	30 (100)
No	0 (0)
Previous radiation dose, n (%)	
> 60Gy	5
45-60Gy	24
< 45Gy	1
Number of previous chemotherapy regimens, n (%)	
0	1 (3.3)
1	2 (6.7)
2	13 (43.3)
3	7 (23.3)
≥ 4	7 (23.3)
Previous chemotherapy regimen, n (%)	
Temozolomide, with concomitant radiotherapy	25 (83.3)
Temozolomide	21 (70.0)
Temozolomide + Cisplatin	4 (13.3)
Temozolomide + Carboplatin	1 (3.3)
Temozolomide + Apatinib	4 (13.3)
Temozolomide + Bevacizumab	2 (6.7)
Bevacizumab	3 (10.0)
Temozolomide + Nimotuzumab	1 (3.3)
Lomustine	1 (3.3)
Nimustine	1 (3.3)
Teniposide	1 (3.3)
Prior bevacizumab, n (%)	
Yes	5 (16.7)
No	25 (83.3)
Tumor dissemination, n (%)	
Intracranial	25 (83.3)
Intracranial and spinal	5 (16.7)
MGMT status, n (%)	
Unmethylated	9 (30)
Methylated	4 (13.3)
Not done/unknown	17 (56.7)
IDH1/2 mutation, n (%)	
Yes	1 (3.3)
No	10 (33.3)
Not done/unknown	19 (63.3)
EIAED	0 (0)
Non-EIAED	30 (100)
Patients alive	4 (13.3)
Patients not progressed	1 (3.3)
Median treatment length (cycles), range	2 (1–11)

*GBM* glioblastoma, *MGMT* O6-methylguanine DNA-methyltransferase, *IDH* isocitrate dehydrogenase, *EIAED* Enzyme-induced anti-epileptic drugs

### Response to treatment

**Fig. 1:**
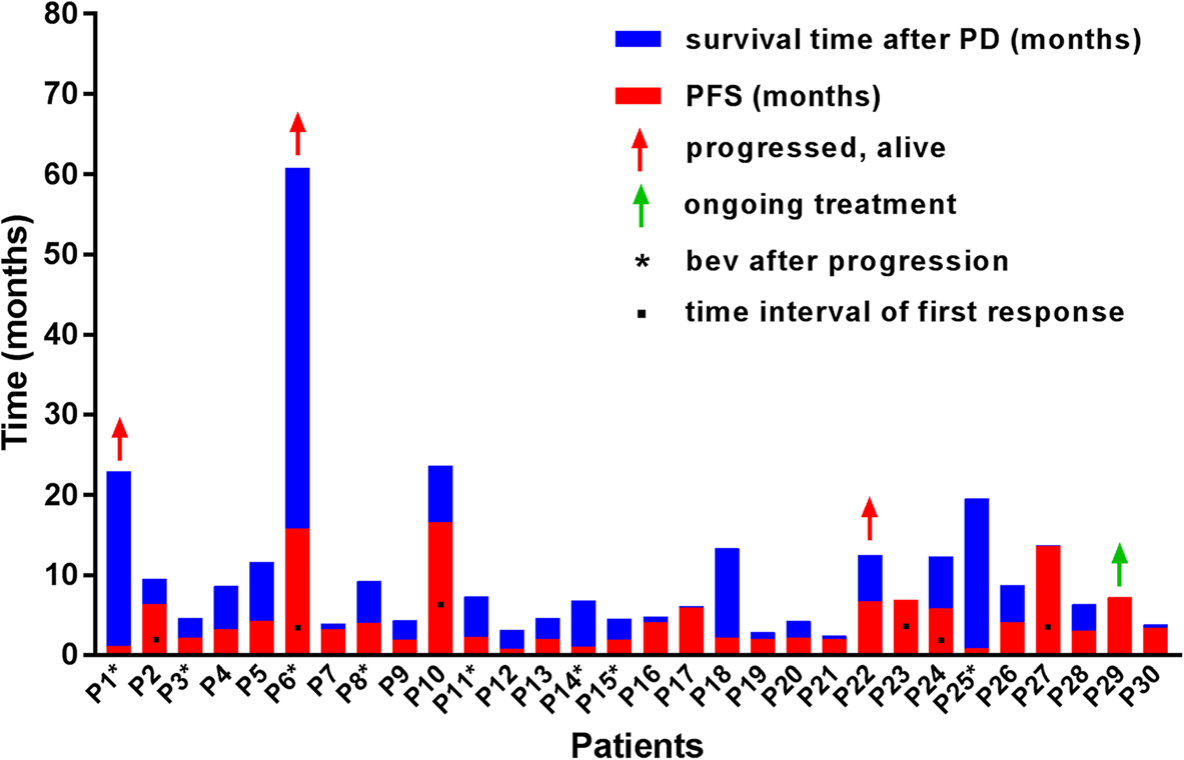
Overview of the theraputic effects. Seven of 30 patients received more than 4 cycles of chemotherapy, including 6 patients got partial response (black spot) and 1 being in treatment (green arrow). The median time interval of first response was 4 (range, 2.0–6.6) months. Three patients got progressed, but not died (red arrow). After progression, 8 patients received bevacizumab treatment (marked with asterisk)

Of the 30 patients, 6 achieved partial response, 11 got stable disease and 13 got progression disease. The ORR was 20% and DCR was 56.7%. Figure 1 summarized the therapeutic effects of all the 30 patients. Seven of 30 patients received more than 4 cycles of chemotherapy, including 6 got partial response and 1 being in treatment. Three patients got progressed, but were still alive (red arrow). After progression, 8 patients received bevacizumab treatment (marked with asterisk).

**Fig. 2:**
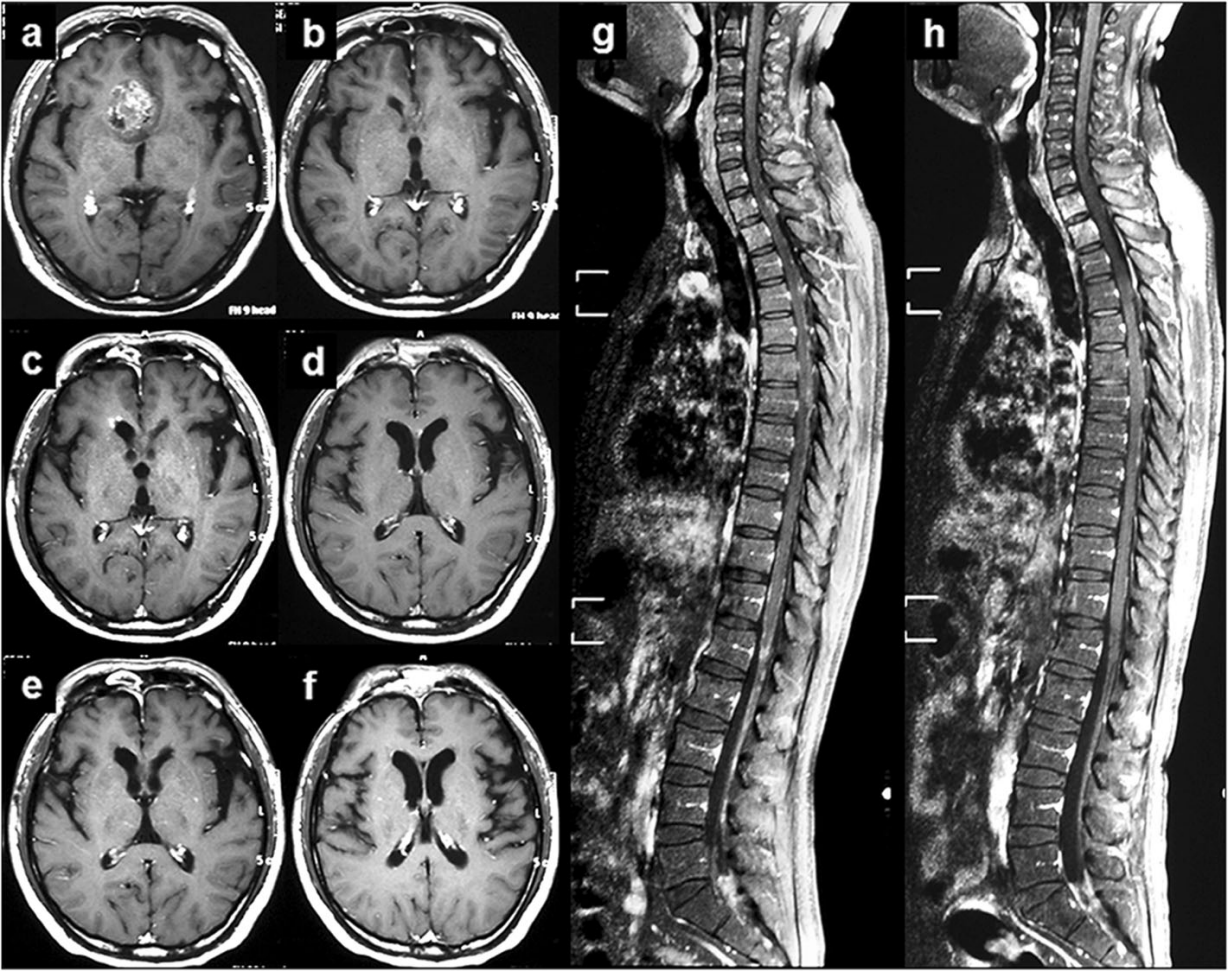
MRI of a typical case before and after treatment. **a** Evidence of a Gadolinium-enhanced lesion in the right frontal lobe before first surgery. **b** After surgery, chemoradiotherapy and adjuvant TMZ-based chemotherapy, the tumor got a complete response. **c**, **d** and **g** Eleven months after initial treatment completion, tumor recurrence was confirmed by MRI, which demonstrated widespread disseminated lesions in the frontal horn of right lateral ventricle, genu of corpus callosum and spine. **e**, **f** and **h** After 4 months of combined chemotherapy, the tumors were significantly decreased

Figure 2 showed the MRI changes of the patient 27. The tumor was located in right frontal lobe at diagnosis (Fig. 2a). After resection, radiotherapy and chemotherapy, the tumor was disappeared (Fig. 2b). However, 11 months after completion of the initial combined treatment, disseminated metastatic tumors occurred at frontal horn of the right lateral ventricles, the genu of corpus callosum and spinal (Fig. 2c, d and g). Then he received chemotherapy with TMZ, CPT-11 and rh-ES. After 4 months, the disseminated tumors were significantly deceased and got a patial response (Fig. 2e, f and h). After 11 cycles, he discontinued the combined chemotherapy. However, 2 months later, he died from cerebral hernia.

### Survival

**Fig. 3:**
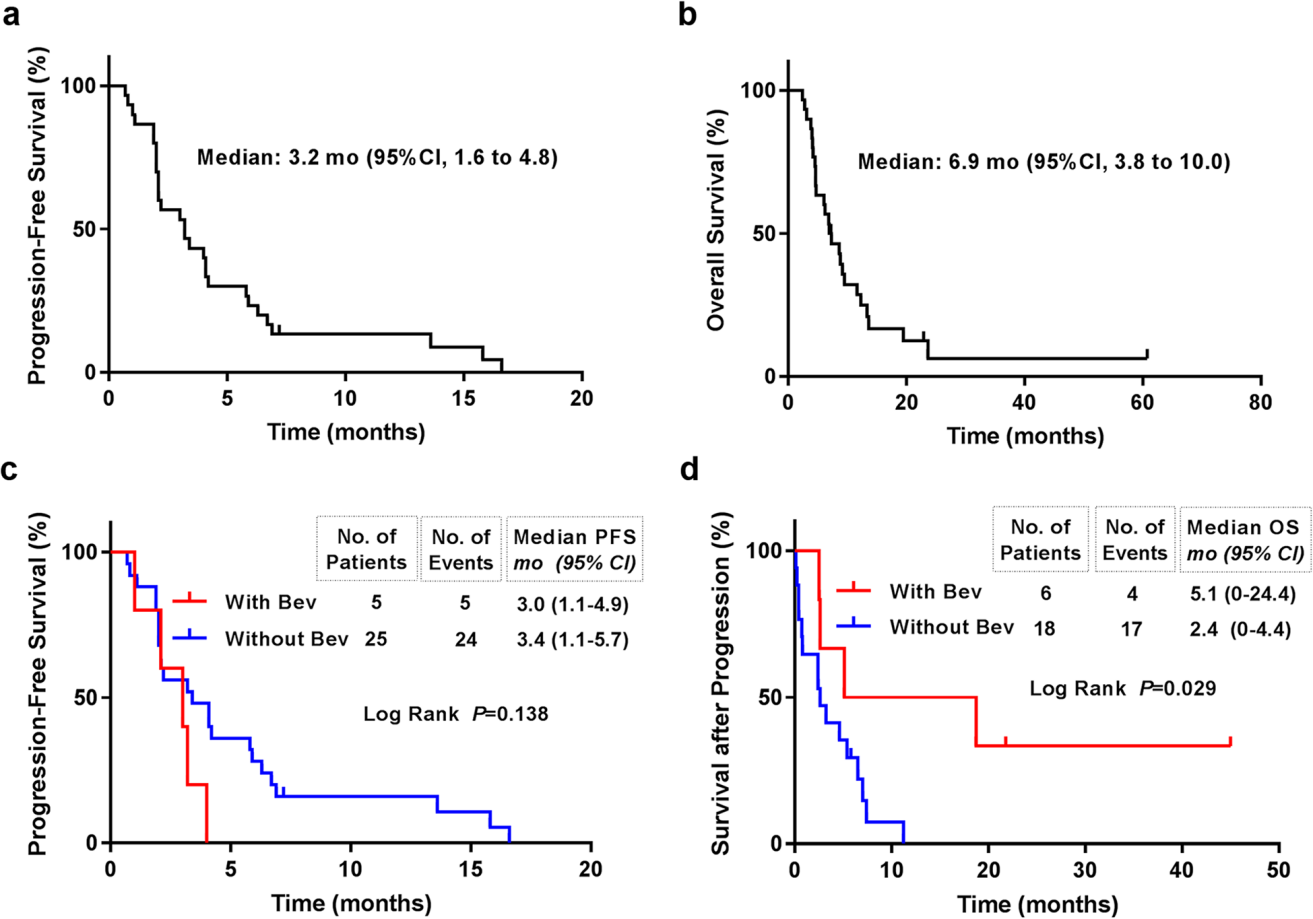
Kaplan-Meier curves of Progression-Free Survival (PFS) and Overall Survival (OS). **a** PFS curve of all the enrolled patients; **b** OS curve of all the enrolled patients; **c** PFS curves of patients who received bevacizumab or not before enrollment. **d** OS curves of patients who received bevacizumab or not after tumor progression. Note: Bev, bevacizumab; mo, months

At the last follow-up (March 31, 2019), 1 of 30 (3.3%) patients were still not progressed and 4 (13.3%) were still alive. The Kaplan-Meier curves of PFS and OS were showed in Fig. 3. The 6 m-PFS was 23.3% (95% CI, 8.2 to 38.4%). The median PFS was 3.2 (95%CI, 1.6 to 4.8) months (Fig. 3a). The 12 m-OS was 28.6% (95% CI, 12.1 to 45.1%). The median OS was 6.9 (95%CI, 3.8 to 10.0) months (Fig. 3b).

The PFS of the 6 patients who got partial response was 5.8, 6.3, 6.9, 13.6, 15.8, 16.6 months, respectively. The median time interval of first response was 4 (range, 2.0–6.6) months (Fig. 1). This demonstrated that the patients could achieve a long PFS once they got radiographic response in about 4 months.

Five of the 30 patients received previous bevacizumab treatment before enrollment. We analyzed the effect of previous bevacizumab on the survival time. The median PFS was 3.0 (95%CI, 1.1 to 4.9) months versus 3.4 (95%CI, 1.1 to 5.7) months (Log rank *p* = 0.138) for the patients with previous bevacizumab treatment or not, respectively (Fig. 3c); the OS was 6.2 (95%CI, 1.7 to 10.7) months versus 8.6 (95%CI, 5.7 to 11.5) months, respectively (Log rank *p* = 0.098). This suggested that previous bevacizumab treatment may decrease the effect of combined chemotherapy though the difference was not statistical significant. This might be due to the small sample size.

After the tumor progression, 14 of the 29 patients received anticancer therapy, such as chemotherapy (12), radiotherapy (1) and surgery (1). Except for the 5 with previous bevacizumab, 6 out of 24 (25%) patients received salvage bevacizumab treatment. The median survival time after progression of the patients with or without bevacizumab was 5.1 (95%CI, 0 to 24.4) months versus 2.4 (95%CI, 0 to 4.9) months, respectively (Log Rank *p* = 0.029) (Fig. 3d). This indicated bevacizumab treatment after progression from combined therapy may prolong the survival time.

We then investigated the relationship between chemotherapeutic effects and MGMT and IDH. The median PFS of the patients with unmethylated or methylated MGMT was 2.1 (95%CI, 1.8 to 2.4) months versus 2 (95%CI, 0 to 4.5) months, respectively (Log Rank, *p* = 0.92). The median PFS of the patients with mutated or wild-type IDH was 0.7 months versus 2 (1.8 to 2.2) months, respectively (Log Rank, *p* = 0.002). However, because more than half patients lost the information of IDH mutation status and MGMT methylation status, the results of the related PFS were not convincing which needed further study to confirm.

### Toxicity

All the 30 patients were available for the safety analysis. There were no deaths from treatment-related toxicity. No one discontinued treatment due to adverse events. The most common adverse events (any grade) were hematologic toxicity (22, 73.3%) and gastrointestinal reactions (11, 36.7%), including nausea and vomiting (8, 26.7%), decreased appetite (6, 20%) and diarrhea (2, 6.7%). The other adverse events included elevated aminotransferase (5, 16.7%), dizziness (2, 6.7%), palpitation (2, 6.7%) and fatigue (1, 3.3%) (Table 2). Twelve out of 30 (40%) patients experienced Grade ≥ 3 adverse events: hematologic toxicity (12, 40%) and elevated aminotransferase (1, 3.3%). Dizziness and palpitation were mostly due to rh-ES and diarrhea was due to CPT-11. Dose reductions of CPT-11 or TMZ were occurred in 5 patients (16.7%). Generally, the treatment was relatively well tolerated.

**Table 2:** The toxicities of the combined chemotherapy of temozolomide, irinotecan and recombinant human endostatin

Toxicity	Any Grade (%)	Grade 3 and 4 (%)
Hematologic	22 (73.3)	12 (40.0)
Leukopenia	22 (73.3)	11 (36.7)
Neutropenia	22 (73.3)	9 (30.0)
Thrombocytopenia	4 (13.3)	3 (10.0)
Lymphocytopenia	2 (6.7)	0 (0)
Nonhematologic		
Nausea and vomiting	8 (26.7)	0 (0)
Decreased appetite	6 (20.0)	0 (0)
Diarrhea	2 (6.7)	0 (0)
Elavated aminotransferase	5 (16.7)	1 (3.3)
Dizziness	2 (6.7)	0 (0)
Palpitation	2 (6.7)	0 (0)
Fatigue	1 (3.3)	0 (0)

## Discussions

### Promising results of this rh-ES-combined chemotherapy

The prognosis of recurrent disseminated glioblastoma is very poor with a mean OS of only 2 to 4 months even after kinds of treatments [13–15]**.** A recent retrospective study was conducted to examine the prognosis of glioma patients with leptomeningeal disease over 15-year period. It was demonstrated that 128 patients with glioblastoma had a median OS of 3.8 (0.1–32.6) months after leptomeningeal disease diagnosis [16]. Improved therapeutic approaches are needed for recurrent glioblastoma with neuraxis dissemination.

In recent years, there are several reports on treatment of adult disseminated glioblastoma. Mandel JJ retrospectively reviewed 36 glioblastoma with leptomeningeal dissemination (including the newly-diagnosed) and assessed the impact of a variety of treatment modalities (hospice or radiation or chemotherapy). The survival time from the dissemination diagnosis was only 3.5 months. A combination of chemotherapy/targeted therapy and radiation had a significantly prolonged survival. However, for recurrent patients, re-radiation was always not recommended due to the dose restriction. In addition, this study did not focus on one chemotherapy regimen and fail to provide an effective therapeutic approach [17]. Most of the other published studies were case reports [18–20]. Okita Y reported a case of leptomeningeal dissemination of recurrent glioblastoma that achieved transient neurological and radiological improvement after chemotherapy with TMZ and bevacizumab (PFS = 2.3 months) and died 5 months after diagnosis of dissemination [18]. In addition, a significant long-term remission (> 2.5 years) of disseminated glioblastoma to bevacizumab was observed in a patient with encephalocraniocutaneous lipomatosis. However, the younger age (32-year-old) might influence the survival. Moreover, the biological differences between this particular glioblastoma in patient with encephalocraniocutaneous lipomatosis and common glioblastoma remain unknown [20].

Although the effect of CPT-11 combined with TMZ or bevacizumab in recurrent glioblastoma was investigated in sevaral studies, the status of dissemination of enrolled patients was not mentioned [21, 22].

The current study demonstrated a prolonged PFS and OS. In this study, after received combined chemotherapy regimen of rh-ES, TMZ and CPT-11, the 6 m-PFS was 23.3%, the median PFS and OS was 3.2 months and 6.9 months, respectively. The longest PFS was 16.6 months. This combined chemotherapy regimen was considered an effective salvage chemotherapy for recurrent disseminated glioblastoma.

### Possible mechanisms underlying the synergistic effects

Different mechanisms, lack of overlapping major toxicities and the potent synergistic effect make these three drugs ideal candidates for combination chemotherapy. CPT-11, a topoisomerase I inhibitor, prevents re-ligation of DNA double strands and inhibits DNA replication, transcription and repair, resulting in tumor cell death. Toxicities of CPT-11 included diarrhea and relatively modest myelosuppression. CPT-11 could cross the blood brain barrier (BBB), which demonstrates excellent central nervous system penetration, but has shown only modest efficacy (6 m-PFS was 15.7%) in patients with recurrent primary glioblastoma [23]. TMZ is a methylating agent that generates O^6^-methylguanine in DNA. TMZ is the standard therapy for patients with malignant glioma with myelosuppression as the main toxicity. Angiogenesis is a significant regulator of glioblastoma growth and tumor vascularity correlates with high-risk disease [24, 25]. Rh-ES is an endogenous broad-spectrum angiogenesis inhibitor, which could inhibit VEGF, cyclin D1, metalloproteinases, c-myc, integrins, and even Wnt signaling, thus inhibiting endothelial cell proliferation and migration, suppressing tumor vascularization and blocking the nutrition and oxygen supply to tumor cells [26–28]. The toxicity of rh-ES was mainly the modest cardiovascular toxicity.

The possible mechanisms underlying the synergistic effects were as follows: (1) Methylation of O^6^-guanine by TMZ may lead to recruitment of topoisomerase I and potentially enhance the probability of inducing CPT-11-mediated damage. (2) The efficacy of gliomas treatments relies on drugs brain distribution through the BBB, a monolayer of endothelial cells. In an in vivo model, TMZ and CPT-11 were shown that they could be transported by ATP-binding cassette B1 (ABCB1), the main efflux transporter expressed at the BBB level, which could influence the efficacy of TMZ and CPT-11 [29]. In a preclinical trial of NSCLC side population cells, rh-ES could prevent the migration of endothelial cells induced by VEGF and ABCB1 and inhibit the angiogenesis [30]. (3) Rh-ES decreases interstitial fluid pressure, promotes vascular normalization, thus induces a pressure gradient across the vasculature and improves drug penetration in tumors [31]. (4) The broad-sperum characteristic of rh-ES could help to reduce drug resistance and act synergistically with other cytotoxic drugs [8]. More direct evidences of the mechanism are needed to investigate in future preclinical studies.

### Postponing the use of bevacizumab and prolonging the survival time

Bevacizumab may also aid in normalizing this disrupted tumor vasculature and improve drug delivery by facilitating uniform distribution of the vasculature [32–34]. Bevacizumab has been proved effective for recurrent glioblastoma.

Nonetheless most patients’ tumors progress after a median time of 3–5 months or even during the bevacizumab administration [5–7]. The reasons of bevacizumab resistance might be the activation of other angiogenic pathways other than VEGF [35, 36]. After progression on bevacizumab, there is no consensus on subsequent therapy, as multiple chemotherapy trials have failed to demonstrate discernible activity for salvage [37, 38]. Postponing the use of bevacizumab might be an alternative strategy.

In this current retrospective study, the patients could get radiographic response in a short time (4 months) and achieve a long PFS once getting response. Moreover, after tumor progression from the combined chemotherapy of rh-ES, TMZ and CPT-11, bevacizumab usage could help to prolong the survival time (5.1 months versus 2.4 months). The current combined regimen did not reduce the sensitivity of tumor to bevacizumab. This study provided an approach to postpone the use of bevacizumab and prolonging the survival time. The combined regimen of rh-ES, TMZ and CPT-11 was an alternative effective regimen prior to bevacizumab in treatment of recurrent disseminated glioblastoma.

## Conclusion

In conclusion, the current study shows rh-ES combining TMZ and CPT-11 is an alternative effective regimen with manageable toxicities in treating recurrent disseminated glioblastoma. However, there are many further studies need to conduct: (1) large-scale prospective trials to investigate the effect of the combined chemotherapy regimens; (2) the mechanism underlying the synergistic effects.

## Data Availability

The datasets used and/or analyzed during the current study are available from the corresponding author on reasonable request.

## Abbreviations

ABCB1: ATP-binding cassette B1
BBB: Blood brain barrier
CPT-11: Irinotecan
DCR: Disease control rate
EIAEDs: Enzyme-inducing antiepileptic drugs
ORR: Objective response rate
OS: Overall survival
PFS: Progression-free survival
rh-ES: Recombinant human endostatin
TMZ: Temozolomide
VEGF: Vascular endothelial growth factor
